# Blind Mesh Assessment Based on Graph Spectral Entropy and Spatial Features

**DOI:** 10.3390/e22020190

**Published:** 2020-02-07

**Authors:** Yaoyao Lin, Mei Yu, Ken Chen, Gangyi Jiang, Fen Chen, Zongju Peng

**Affiliations:** Faculty of Information Science and Engineering, Ningbo University, No. 818, Ningbo 315211, China; linyaoyao0203@126.com (Y.L.); chenken@nbu.edu.cn (K.C.); chenfen@nbu.edu.cn (F.C.); pengzongju@nbu.edu.cn (Z.P.)

**Keywords:** blind mesh quality assessment, graph signal processing, graph spectral entropy features, spatial features

## Abstract

With the wide applications of three-dimensional (3D) meshes in intelligent manufacturing, digital animation, virtual reality, digital cities and other fields, more and more processing techniques are being developed for 3D meshes, including watermarking, compression, and simplification, which will inevitably lead to various distortions. Therefore, how to evaluate the visual quality of 3D mesh is becoming an important problem and it is necessary to design effective tools for blind 3D mesh quality assessment. In this paper, we propose a new Blind Mesh Quality Assessment method based on Graph Spectral Entropy and Spatial features, called as BMQA-GSES. 3D mesh can be represented as graph signal, in the graph spectral domain, the Gaussian curvature signal of the 3D mesh is firstly converted with Graph Fourier transform (GFT), and then the smoothness and information entropy of amplitude features are extracted to evaluate the distortion. In the spatial domain, four well-performing spatial features are combined to describe the concave and convex information and structural information of 3D meshes. All the extracted features are fused by the random forest regression to predict the objective quality score of the 3D mesh. Experiments are performed successfully on the public databases and the obtained results show that the proposed BMQA-GSES method provides good correlation with human visual perception and competitive scores compared to state-of-art quality assessment methods.

## 1. Introduction

As three-dimensional (3D) meshes can provide very realistic visual information for users, the applications of 3D mesh in intelligent manufacturing, digital animation, virtual reality, digital cities, and other fields are becoming more and more widespread [1]. However, 3D meshes can be compressed, simplified, embedded with watermarks, etc., in practical applications [2,3], which inevitably leads to visual distortions of the 3D mesh. Therefore, how to better assess 3D mesh visual quality of the 3D mesh to provide a standard for measuring the performance of compression, simplification and other technologies is a critical problem that needs to be solved [4]. In fact, 3D mesh quality assessment can be divided into subjective and objective quality assessment methods. Subjective assessment results are close to the subjective visual perception of human eyes, but this method is time-consuming, laborious, too expensive, and prone to cause errors due to artificial uncertainties. Therefore, it is necessary to propose an objective quality assessment method that has good consistency with the subjective perception of human eyes.

Generally, 3D mesh quality assessment (MQA) methods can be divided into full-reference (FR), reduced-reference (RR), and non-reference (NR), with the NR method also being referred to as the blind method [5]. Up until now, most of the existing objective 3D MQA methods have mainly been FR or RR. Among the FR methods, Hausdorff Distance (HD) [6] calculates and compares the absolute distance between the distorted and reference 3D meshes, and does not pay attention to the variability of 3D meshes, so the consistency with human subjective visual perception is poor. Karni et al. [7] used the information of vertex coordinate positions and combined the geometric Laplacian operator of the vertex coordinates to calculate the geometric distance between the distorted and the reference 3D meshes. Later, Karni’s method was improved by Sorkine et al. [8]; they gave greater weight to the geometric Laplacian operator. Corsini et al. [9] proposed a method based on global roughness variation to evaluate the visual quality of the watermarked 3D mesh, in which the roughness of the 3D mesh was defined as the variance of the difference between reference 3D mesh and its smoothed version.

Gelasca et al. changed the definition of roughness, that is, they adopted the variance of the dihedral angles between adjacent faces in a multi-resolution way to represent the roughness, and proposed a method called as 3DWPM2 [10]. Lavoué et al. [11] proposed a mesh structural distortion measure (MSDM) method; they extended the Structure Similarity index of 2D images (SSIM) to the 3D meshes, compared the difference between the reference and the distorted 3D meshes by comparing three characteristics of curvature. Later, Lavoué et al. extended the work on the basis of [11], proposing MSDM2 [12], which mainly considered the problem of multi-scale. Vasa et al. [13] introduced the visual concealment effect and proposed a mesh error based on the dihedral angle (DAME) method. Among the RR methods, Wang et al. [14] proposed the fast mesh perceptual distance (FMPD) method, which is based on local roughness, that is, local roughness is analyzed, and local roughness and overall roughness are adjusted in the method. Abouelaziz et al. [15] proposed two RR MQA methods (called as KLD Gamma and KLD Weibull, respectively) using the statistical distribution of dihedral angles between the distorted and reference 3D meshes. With respect to the NR method, Abouelaziz et al. [16] defined an index for calculating the surface roughness and proposed a blind mesh quality assessment (BMQA) method (called as NR-SVR) based on the Gamma statistical model. Abouelaziz et al. [17] proposed a blind MQA (BMQA) method using a convolutional neural network (CNN) based on the extraction of visual representative features.

The methods mentioned above mostly belong to FR or RR, and need to refer to the full or partial information of the original 3D mesh to calculate the geometric distance or extract some features containing perceptual information to evaluate the 3D mesh visual quality. However, in most cases, the reference 3D mesh is not available. Therefore, it is crucial to develop BMQA methods. In addition, the features extracted by the various methods mentioned above are mainly the spatial features, such as the Hausdorff distance, curvature, dihedral angle, and so on [18]. However, the similarity and correlation between the vertices of the 3D mesh cannot be well reflected in the spatial features, and the results show that the consistency of these methods with human visual perception needs to be improved. Therefore, it is important to explore new features that can well describe the characteristics of the distorted 3D mesh in other transform domains. Recently, there has been massive progress in research on sensor networks, traffic transportation networks, communication networks and biological brain networks [19], and the theory of graph signal processing (GSP) has been proposed [20]. Related studies have shown that these GSP analysis tools can be used to solve various irregular signal processing problems in the spectral domain [21,22]. Therefore, in this paper, Graph Fourier transform (GFT) [23] is firstly used to convert the signal in the spatial domain into the graph spectral domain. Then, efficient graph spectral features are extracted and adopted to sparsely represent the distortion of the 3D mesh to reveal the underlying shape characteristics of the 3D mesh in the graph spectral domain. Finally, some improved spatial features are combined and a new BMQA method is proposed. The main contributions in this paper are summarized as follows:

(1) Most of the existing 3D MQA methods are not blind. We propose a new BMQA method based on Graph Spectral Entropy and spatial features, referred to as BMQA-GSES. New features in the graph spectral domain and spatial domain are defined and extracted for BMQA. The graph spectral features of 3D mesh can reveal the underlying shape information, while the spatial features of the 3D mesh can simulate the external information of the 3D mesh that can be directly perceived by human eyes.
(2) Inspired by GSP, the Gaussian curvature signal is transformed from the spatial domain to the graph spectral domain by GFT in the proposed method. In addition, the signal smoothness and information entropy of amplitude features under different frequency components are then extracted in the graph spectral domain as underlying shape features of 3D mesh.
(3) Considering that the concavity, convexity and the structural information of the distorted 3D mesh will change, four spatial features are combined on the base of extracting the graph spectral features.

The rest of this paper is organized as follows. Section 2 introduces the motivation of this paper. Section 3 describes the details of the proposed BMQA-GSES method. Section 4 analyzes the performances of all the features of the proposed method and compares it with state-of-art quality assessment methods. Section 5 concludes the paper.

## 2. Motivation

**Figure 1:**
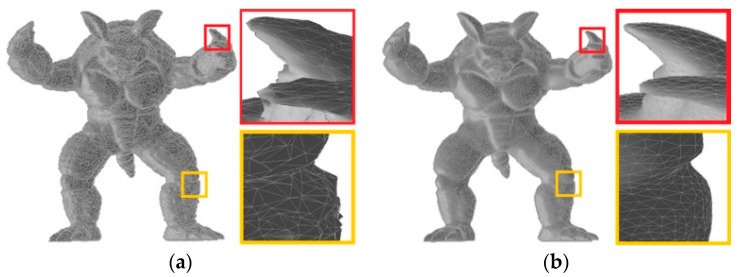
Two types of distorted 3D meshes. (**a**) Distorted 3D mesh with random noise, (**b**) distorted 3D mesh smoothed by smoothing filter.

The shape representation of 3D mesh depends largely on the coordinate of the vertices of the 3D mesh and the distribution of triangular structures. Figure 1 shows two types of distorted 3D meshes, i.e., the distorted 3D mesh with random noise in Figure 1a and the distorted 3D mesh smoothed by a smoothing filter in Figure 1b. From Figure 1, it can be seen that the distorted 3D meshes change significantly in the spatial domain. On the one hand, the surface of the model in Figure 1a is significantly more uneven, while the surface of the 3D model in Figure 1b is smoother. On the other hand, the coordinate of every vertex and the distribution of triangular structure of the distorted mesh will change after being processed in various ways, resulting in distortion of the overall shape of the 3D mesh. At present, most 3D MQA methods focus on spatial features, and they work to a certain extent because the human eye can be prone to directly perceiving changes in the 3D mesh in the spatial domain. However, the similarity and correlation between the vertices of the 3D mesh cannot be well reflected in the spatial features only, and the results show that the consistency of these methods with human visual perception needs to be improved. Therefore, it is important to explore more effective features that can better describe the characteristics of the distorted 3D mesh in graph spectral domains.

Recently, there has been massive progress in research on sensor networks, traffic transportation networks, communication networks, and biological brain networks, which has caused a lot of researchers to pay attention to the irregular signals and data of these networks. In general, irregular signals can be denoted by the graphs of vertices, edges, and weights [24]. Therefore, graph signal processing (GSP) theory was proposed to deal with the irregular graph signals [20]. In this study field, the regular time-domain or spatial domain operators, theorems and tools can also be extended to the vertex domain, including Fourier transform, frequency-selective filter and vertex-frequency analysis, etc. Some GSP tools can be used to solve various irregular signal processing problems [25].

**Figure 2:**
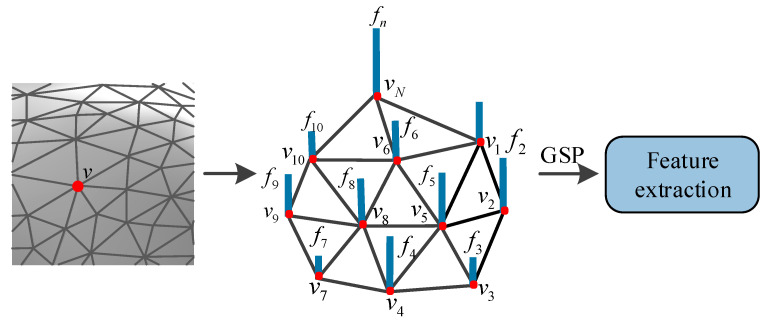
Feature extraction of irregular graph signal based on GSP theory.

In fact, the signal for the vertices of 3D mesh can be regarded as an irregular graph signal, which indicates that GSP theory can provide a new way to describe the distortion of 3D mesh. As shown in Figure 2, the relevant features of 3D mesh can be described as one-dimensional signals on the vertices, which can be transformed by GSP theory into other domains, and then the new features can be extracted from these domains. Therefore, the purpose of this paper is to transform the irregular graph signal in the spatial domain of the 3D mesh into the graph spectral domain through the Graph Fourier transform (GFT), so as to extract some new graph spectral features for appropriate graph spectral features can sparsely represent the distortion of the 3D mesh and reveal the underlying shape characteristics of the 3D mesh. In addition, for the sake of reflecting the distorted 3D mesh more effectively, the improved spatial features are combined to establish a new BMQA method.

## 3. The Proposed BMQA-GSES Method

**Figure 3:**
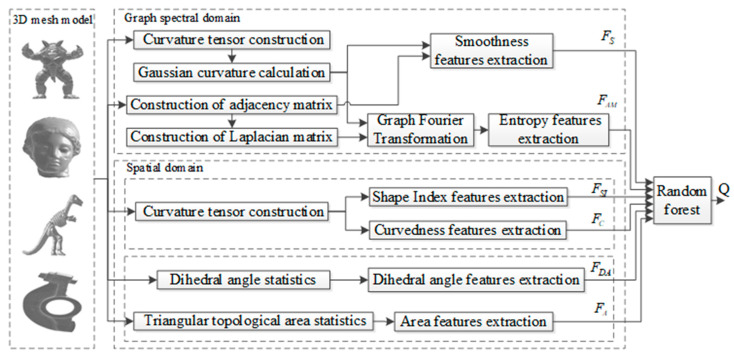
Framework of the proposed BMQA-GSES method.

Based on the analysis above, a new BMQA method, called as BMQA-GSES, is proposed and its specific framework is shown in Figure 3. The method proposed in this paper mainly includes two aspects: graph spectral features and spatial features. In the graph spectral domain, the Gaussian curvature signal of the 3D mesh is transformed to the graph spectral domain by GFT. Then, the signal smoothness and the information entropy of amplitude features under different frequency components are extracted to describe the characteristics of the distorted 3D mesh. In the spatial domain, features with concave, convex and structural information of 3D mesh are extracted. Specifically, the features of shape index and curvedness are used to measure the concave and convex characteristic of the 3D mesh, while the features of dihedral angle and the distribution of the triangular topology structure of 3D mesh are used to measure structural characteristic of the 3D mesh. All these graph spectral and spatial features are fused and learned along with the subjective scores using random forest regression to build the quality prediction model, BMQA-GSES, of the 3D mesh.

### 3.1. Graph Spectral Features Analysis

Appropriate graph spectral features can sparsely represent the distortion of 3D mesh and reveal the underlying shape characteristics of 3D mesh. Therefore, the spectral features are extracted prior to describe information that cannot be directly perceived by human eyes. To be specific, the Gaussian curvature signal is firstly defined on the weighted graph of the 3D mesh, because it has been proved that the Gaussian curvature has good performance in the spatial domain [26] and it has also been found that the performance can be greatly improved when Gaussian curvature signal is transformed from the spatial domain to the graph spectral domain by GFT.

**Figure 4:**
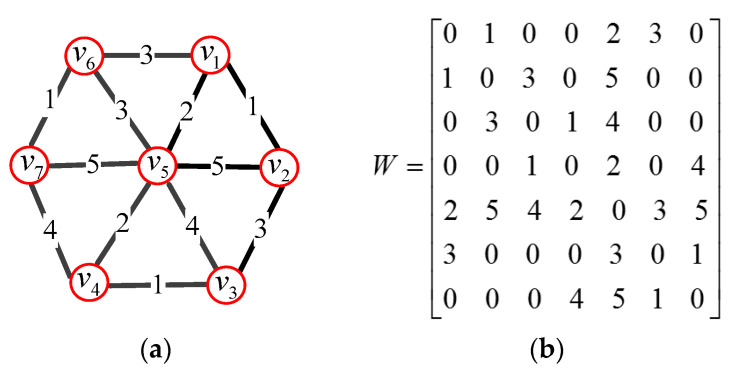
A weighted graph and its corresponding weighting matrix. (**a**) An example of a weighted graph; (**b**) Weighting matrix.

To analyze the graph spectral features of the 3D mesh, the construction of weighted graph is the premise. The 3D mesh can be represented by undirected, connected, weighted graph G = {*v*, *ε*, *W*}, where *v* represents vertices of 3D mesh, *ε* represents edges of 3D mesh, and **W** is weighted adjacency matrix of 3D mesh. If there is an edge *e* = (*i*, *j*) connecting vertices *v_i_* and *v_j_*, the entry *W_i_*_,*j*_ represents the weight of the edge; otherwise, *W_i_*_,*j*_ = 0. Figure 4a shows an example weighted graph, and its corresponding matrix is shown in Figure 4b.

In this paper, the weight, *W_i_*_,*j*_, of an edge connecting vertices *v_i_* and *v_j_* is expressed by a threshold Gaussian kernel weighting function

(1) $$W_{i,j}=\left\{ \begin{array}{cc} \exp(-\frac{{\left[dist(v_{i},v_{j})\right]}^{2}}{2\sigma^{2}}) & \mathrm{if}\;(v_{i},v_{j})\in \varepsilon \\ 0 & \text{otherwise} \end{array}\right.$$

where *dist*(*i*, *j*) represents a distance between the vertices *v_i_* and *v_j_*, and σ is the variance of the distance. 

The smoothness can reveal the intrinsic structure of the graph signal to a certain extent. Let *f_GC_* be the Gaussian curvature signal on the weighted graph, then the smoothness can be defined to describe the intrinsic structural characteristics of the graph signal. The edge derivative of a signal *f_GC_* with respect to edge e=(i,j) at the vertex *v_i_* is defined as

(2) $${\frac{\partial f_{GC}}{\partial e}|}_{i}=\sqrt{W_{i,j}}\left[f_{GC}(v_{j})-f_{GC}(v_{i})\right]$$

where *f_GC_* (*v_i_*) is the Gaussian curvature signal at *v_i_*, and is expressed as

(3) $$f_{GC}(v_{i})=k_{max}(v_{i})\times k_{min}(v_{i})$$

where *k_max_* (*v_i_*) and *k_min_* (*v_i_*) represent the maximum and minimum values of the principal curvature of *v_i_*, respectively.

Then, the local variation at *v_i_* can be computed by

(4) $$\begin{array}{c} {\left\|\nabla_{i}f_{GC}\right\|}_{2}={\left[{\sum_{e\in \varepsilon \;s.t.e=(i,j)\;\mathrm{for}\;\mathrm{some}\;j\in v}\left({\frac{\partial f_{GC}}{\partial e}|}_{i}\right)}^{2}\right]}^{\frac{1}{2}}\\ ={\left[{\sum_{j\in N_{i}}W_{i,j}\left[f_{GC}(v_{j})-f_{GC}(v_{i})\right]}^{2}\right]}^{\frac{1}{2}} \end{array}$$

Thus, the smoothness *F_S_* of the Gaussian curvature signal *f_GC_* on the weighted graph *G* can be defined by

(5) $$F_{S}=\sum_{i\in v}{\left\|\nabla_{i}f_{GC}\right\|}_{2}^{1}=\sum_{i\in v}{\left[{\sum_{j\in N_{i}}W_{i,j}\left[f_{GC}(v_{j})-f_{GC}(v_{i})\right]}^{2}\right]}^{\frac{1}{2}}$$

**Figure 5:**
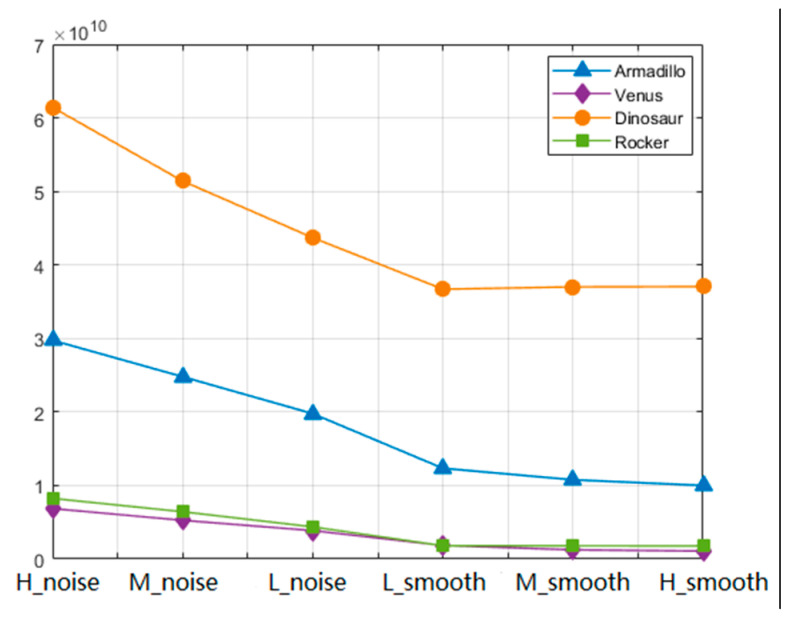
Comparison results of smoothness *F_S_* of four 3D meshes.

**Figure 6:**
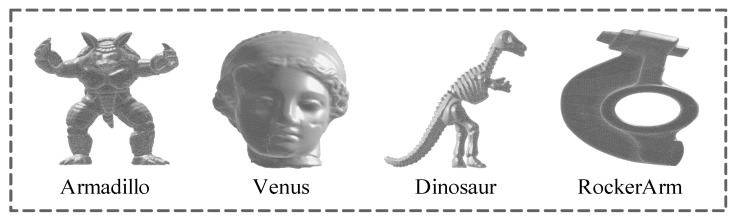
Four kinds of 3D mesh from the public LIRIS/EPFL general-purpose database [9].

To illustrate the effectiveness of smoothness in 3D mesh, Figure 5 shows the comparison results of the smoothness of Gaussian curvature signals corresponding to the different distortion degrees of four 3D meshes. As shown in Figure 6, four 3D meshes are derived from the public LIRIS/EPFL general-purpose database [9]. Firstly, as far as the 3D mesh is concerned, the structures of “Dinosaur” and “Armadillo” are relatively rough. Therefore, the smoothness calculated by these two 3D meshes is relatively large, in other words, the global variance of the model is relatively large, which is consistent with the performance of human visual perception. Secondly, for each particular model, the smoothness decreases with the decrease of roughness. Therefore, the smoothness of the Gaussian curvature signal can be better used to simulate the distortion of 3D mesh. Finally, although the relevant work has confirmed that Gaussian curvature can well reflect the concave and convex information of the model in spatial domain, Gaussian curvature is calculated independently based on each isolated vertex, which cannot accurately measure the correlation and similarity between the vertices of the model, and the smoothness of the signal can exactly solve this problem. Therefore, the smoothness *F_S_* of the signal *f_GC_* is adopted to describe the distorted 3D mesh in the proposed method. In general, the irregular signals are denoted by the graphs of vertices, edges, and weights. In this research area, the conventional time-domain or spatial domain operators, theorems and tools are extended to the vertex domain, including Fourier transform, frequency-selective filter and vertex-frequency analysis etc. The theoretical and practical tools for this GSP analysis can be adopted to solve various irregular signal processing problems [23]. Therefore, in the proposed method, the GFT is used to transform the irregular Gaussian curvature signal from spatial domain into graph spectral domain, and then the relevant graph spectral features are extracted to reveal the underlying shape information of 3D mesh and describe the distortion of 3D mesh.

**Figure 7:**
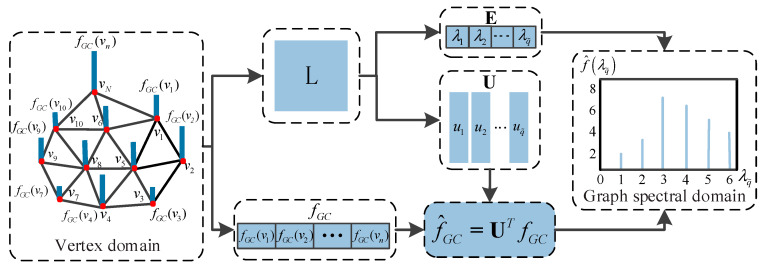
Transformation of graph signal from spatial domain into graph spectral domain.

Figure 7 depicts the transformation of the graph signal from spatial domain into graph spectral domain, and the specific description is given in the supplementary section at the end of this section. For a 3D mesh, its Laplacian matrix **L** is defined as follows

(6) L=D−W

where **W** is the weight matrix, **D** is the degree matrix that can be calculated as follows

(7) $$D_{i,i}=\sum_{j=1}^{N}W_{i,j}$$

L is a sparse, symmetric, positive and semi-definite matrix, and has $\tilde{q}$ non-negative eigenvalues {*λ_q_*; *q* = 0, 1, …, *q*, …, $\tilde{q}$), and $0\leq \lambda_{1}\leq \lambda_{2}\leq \cdots \leq \lambda_{\tilde{q}}$. Thus, $\sigma (L)=\{\lambda_{0},\lambda_{1},\cdots ,\lambda_{\tilde{q}}\}$ represents the frequency component of the whole spectrum. The problem of eigenvalue decomposition can be solved by many methods. In our method, the Lanczos method [27] is adopted to compute the eigenvectors **U** of sparse matrices **L**, where $U=[u_{1},u_{2},u_{3},\cdots ,u_{\tilde{q}}]$.

The classical Fourier transform can be expressed by

(8) $$\hat{f}(\xi )=\langle f,e^{2\pi i\xi t}\rangle =\int_{\mathbb{R}}f(t)e^{-2\pi i\xi t}dt$$

where ξ denotes frequency.

Analogously, the GFT ${\hat{f}}_{GC}$ of the graph signal of any function $f_{GC}\in {\mathbb{R}}^{N}$ defined at the vertice can be expressed as

(9) $${\hat{f}}_{GC}(\lambda_{q})=\sum_{i=1}^{N}f_{GC}(v_{i})\;u_{q}^{*}$$

where eigenvector $u_{q}^{*}$ is a conjugate form of eigenvector $u_{q}$.

In classical Fourier analysis, eigenvalues ${\left\{{\left(2\pi \xi \right)}^{2}\right\}}_{\in \mathbb{R}}$ in (8) carry a specific concept about frequency, that is, for ξ close to zero (low frequencies), the related complex exponential eigenfunctions are relatively smooth and oscillate slowly, while for *ξ* far away from zero (high frequencies), the related complex exponential eigenfunctions oscillate faster. Similarly, in graphical signals, the graph Laplacian eigenvalues and the graph Laplacian eigenvectors provide a similar concept of frequency. For the connected weighted graph constructed by 3D meshes, the Laplacian eigenvectors of the graph associated with low frequency changes relatively slowly in the graph, that is, if an edge connected by two vertices has a large weight, the values of the eigenvectors at those positions may be similar, which means that these locations of the model are relatively smooth. The eigenvectors associated with larger eigenvalues vary more dramatically, and the values on the vertices which are connected by these edges with larger weights are more likely to be different, which also means that these locations of the model are relatively rough. Therefore, the graph spectral features are adopted to describe the roughness of the 3D mesh. Related studies have shown that the information entropy can be used to analyze the characteristics of signals [28,29], and it has been found that information entropy of amplitudes corresponding to the different frequency components can efficiently and concisely reflect the distortion of 3D mesh in this paper. Therefore, in the proposed method, the information entropy of amplitudes of the signal *f_GC_* in the graph spectral domain is used as the graph spectral feature, ***F_AM_***, reflecting the distortion of the 3D mesh, expressed as follow

(10) $$F_{AM}=-\sum_{q=1}^{\tilde{q}}P({\hat{f}}_{GC}(\lambda_{q}))\log P({\hat{f}}_{GC}(\lambda_{q}))$$

where *P* represents the probability that the amplitude of signal *f_GC_* appearing in the spectral domain.

As a supplement, the process of graph signal transformation from vertex domain to graph spectral domain is described in detail in this part. The specific steps are as follows:

(1) The Gaussian curvature on each vertex of the 3D mesh is firstly calculated, denoted as signal $f_{GC}(v_{i})$;
(2) The weighted graph *G* = {*v*,*ε*,***W***} is then used to represent 3D mesh, where *v* represents vertices of 3D mesh, *ε* represents edges of 3D mesh, and **W** is weighted adjacency matrix of 3D mesh;
(3) The graph Laplacian matrix **L** can be calculated from (6) and (7). The graph Laplacian matrix is sparse and has very large dimensions because the 3D mesh is composed of a lot of vertices. Therefore, the Lanczos method is used to calculate the eigenvectors **U** and eigenvalues **E** of the sparse matrix **L**;
(4) The Gaussian curvature signal is transformed by GFT based on the obtained eigenvector **U**, i.e., (9). Since there is a one-to-one correspondence between the eigenvalues and the eigenvectors, the corresponding amplitude ${\hat{f}}_{GC}$ of each eigenvalue can be obtained. The eigenvalues of the GFT replace the concept of frequency in the classical Fourier transform, so the eigenvalues and the corresponding amplitudes constitute the graph spectrum of 3D mesh.
(5) The information entropy of amplitudes under different frequency components is extracted to describe the characteristics of 3D mesh in the graph spectral domain.

### 3.2. Spatial Features Analysis

From Figure 2, it can be found that the distorted 3D mesh will vary greatly in the spatial domain. The surface of the distorted 3D mesh with random noise is uneven, while the surface of the distorted 3D mesh smoothed by a smoothing filter is very smooth. Therefore, in the case of considering the graph spectral features, some excellent spatial features are also combined in this paper. In spatial domain, the features with concave, convex and structural information of 3D mesh are extracted to simulate human visual perception. Specifically, the features of shape index and curvedness are used to measure the concave and convex characteristic of the 3D mesh, while the features of dihedral angle and the distribution of the triangular topology structure of 3D mesh are used to measure structural characteristic of the 3D mesh.

#### 3.2.1. Concave and Convex Feature Analyses

It is obvious that the roughness of 3D mesh has a great correlation with the visual quality of 3D mesh. The surface of rough 3D mesh is often uneven, while the surface of smooth 3D mesh tends to be relatively smooth. Thus, these features of shape index and the curvedness are used to describe the degree of the roughness of 3D mesh to further predict the objective quality score of 3D mesh.

**Figure 8:**
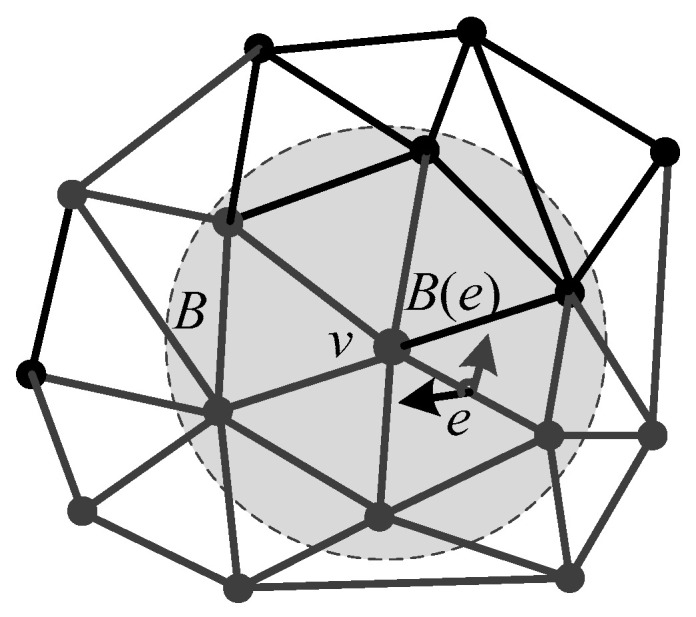
Geometric elements used to compute the curvature tensor.

For a model composed of discrete surfaces, such as 3D mesh, the coordinate positions of the discrete vertices and the topological structure between the discrete vertices are used to calculate the approximate value of the curvature of the discrete surface. At present, there are many curvature estimation methods for 3D mesh. Among them, the normal cycle theory [30] is used in this paper to estimate curvature tensor for its high accuracy. Then, the curvature tensor *T*(*v*) of any vertex *v* in domain *B* can be expressed as

(11) $$T(v)=\frac{1}{\left|B\right|}{\sum_{edges\;e}\beta (e)\left|e\cap B\right|\bar{e}\;\bar{e}}^{T}$$

where *|B|* is the area of *B*, *e* is the edge that is completely or partially contained within the domain *B*, *β*(*e*) is the angle between the normal vectors of two adjacent triangular faces to the edge *e*, *|e∩B|* is the length of the edge *e* located in *B*, and $\bar{e}$ and ${\bar{e}}^{T}$ are the unit vector in the direction of *e* and its transpose, respectively, as shown in Figure 8. In the proposed method, *B* is set to be the 1-ring of each vertex *v*.

The largest eigenvalue, *k_max_*, and the smallest eigenvalue, *k_min_*, can be obtained by constructing the curvature tensor, *T*(*v*). *k_max_* and *k_min_*, respectively, represent the maximum principal curvature and minimum principal curvature of the vertex. Then, the shape index, *SI*, and the curvedness, *C*, can be expressed as follows

(12) $$SI=-\frac{2}{\pi }\arctan\frac{k_{max}+k_{min}}{k_{max}-k_{min}}$$

(13) $$C=\frac{\sqrt{k_{min}^{2}+k_{max}^{2}}}{2}$$

**Figure 9:**
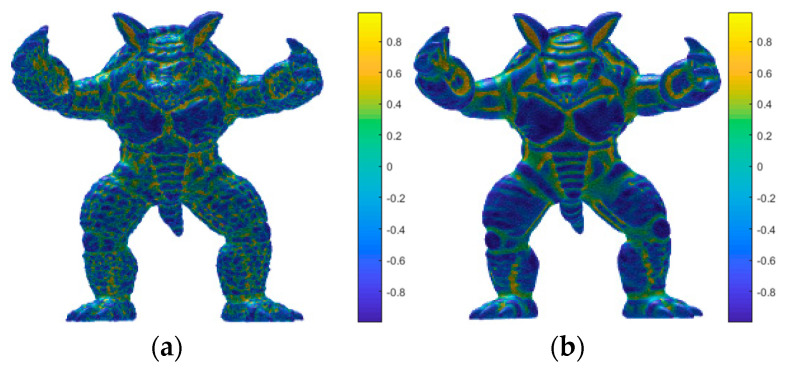
Visual map of the shape index in the vertex domain. (**a**) Distorted 3D mesh with the distortion of random noise, (**b**) distorted 3D mesh with the distortion of smooth.

Figure 9 shows visual maps of the shape index in the vertex domain of the armadillo model. Figure 9a depicts a distorted 3D mesh with the distortion of random noise, while Figure 9b depicts a distorted 3D mesh with the distortion of smooth. In addition, the color scale from bottom to top represents that the value of shape index is getting larger and larger. Obviously, the visual mapping of the model in Figure 9a,b are significantly different, because the surface of the distorted 3D mesh in Figure 9a is very rough and the surface of the distorted 3D mesh in Figure 9b is too smooth. In addition, the shape indexes of different distortion types of different levels are listed in Table 1. It can be concluded that the shape index can well measure the roughness of the 3D mesh surface, and it has good consistency with human visual perception. To improve the efficiency of the proposed method, the generalized Gaussian distribution (GGD) are used to extract the estimated parameters (μ, σ, α) of shape index distribution curve instead of a lot of original data to represent shape index characteristic, then, the final shape index feature vector is obtained by

(14) $$F_{SI}=[SI_{\mu },SI_{\sigma },SI_{\alpha }]$$

where *SI_µ_*, *SI_σ_* and *SI_α_* represent the mean, variance and scale parameter of shape index, respectively.

**Figure 10:**
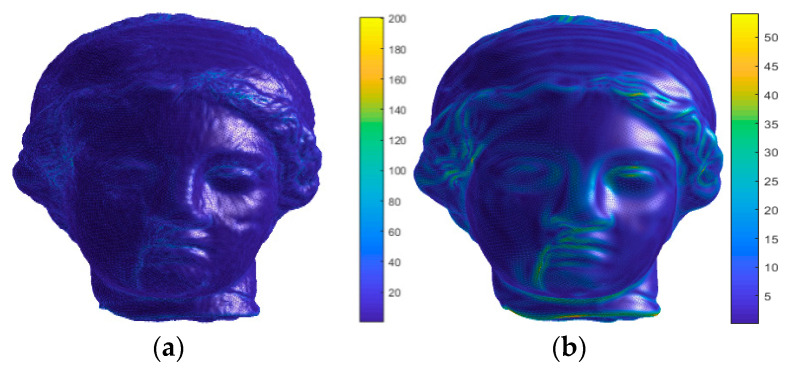
Visual map of the curvedness in the vertex domain. (**a**) Distorted 3D mesh with the random noise distortion, (**b**) distorted 3D mesh with the smooth distortion.

Figure 10 shows visual maps of the curvedness in the vertex domain of the Venus model. Figure 10a is a distorted 3D mesh with the distortion of random noise, while Figure 10b is a distorted 3D mesh with the distortion of smooth. In addition, the color scale from bottom to top represents that the value of curvedness is getting larger and larger. It is obvious that there are extremely different on the curvedness of the distorted 3D mesh in Figure 10a,b. On the one hand, the curvature of the most vertices in Figure 10a is larger than the corresponding vertices in Figure 10b. On the other hand, the curvature of some rough areas such as the hair is larger than the smooth areas such as the cheek, which can indicate that the curvedness can also measure the roughness of the 3D mesh surface, and it also maintains good consistency with the subjective perception of human eyes. Similarly, the GGD are adopted to extract the estimated parameters (μ, σ, α) of curvedness distribution curve to represent curvedness characteristic in this part, then, the final curvedness feature vector is obtained by

(15) $$F_{C}=[C_{\mu },C_{\sigma },C_{\alpha }]$$

where *C_μ_*, *C_σ_* and *C_α_* represent the mean, variance and scale parameter of curvedness.

#### 3.2.2. Structural Feature Analyses

3D mesh model is composed by a lot of vertices and triangular topological surfaces. Compared with the reference 3D mesh, the triangular topological structure of the distorted 3D mesh will change significantly. Thus, two steps are taken in this paper. The first is to count dihedral angle information of the triangular topology of the 3D mesh, the second is to calculate the area of each triangular topology of the 3D mesh. In the first step, the normal vector *n_t_* of each face of the 3D mesh is calculated, and then the dihedral angles of two adjacent faces *t*_1_, *t*_2_ can be obtained by following formula.

(16) $$D_{t_{1},t_{2}}=\arccos(n_{t_{1}}\cdot n_{t_{2}})$$

where $n_{t_{1}}$,$n_{t_{2}}$ represents the normal vector of two adjacent faces *t*_1_, *t*_2_, respectively.

In this part, the mean and variance of dihedral angles are calculated as the structural features to describe deviation degree of the triangular topology of the model, and the dihedral angles feature vector, *F_DA_*, in spatial domain is expressed as

(17) $$F_{DA}=[D_{\mu },D_{\sigma }]$$

where *D_μ_* and *D_σ_* denote the mean and variance parameters of the dihedral angles, respectively. 

In fact, rough 3D meshes usually need a lot of small-area triangular topologies to present highly curved surface shapes, while smooth 3D meshes allow large-area triangular topologies to present a relatively flat surface shapes. Therefore, we perform histogram statistics on the triangular topological area of 3D mesh, and the GGD are adopted to extract the estimated parameters (*μ*, *σ*, *α*) of triangular topological area to represent triangular topological area feature, and the rough feature vector, *F_A_*, is expressed as

(18) $$F_{A}=[A_{\mu },A_{\sigma },A_{\alpha }]$$

where *A_μ_*, *A_σ_* and *A_α_* represent the mean, variance and scale parameter of triangular topological area, respectively. In addition, the triangular topological area can be obtained by a common mathematical formula for calculating the area of a triangle.

### 3.3. Pooling Strategy

Random forest (RF) regression is used to fuse multi-dimensional features in the proposed BMQA-GSES method. RF is a machine learning algorithm with fast training speed that is not easy to overfit. Each decision tree in the forest can judged independently, and the mean value of all the decision trees is taken as the final output of the RF [31].

In this paper, a set of 13-dimensional feature vectors are extracted to jointly predict the quality of the distorted 3D mesh. In the process of training, the extracted features and their corresponding subjective scores of the training 3D meshes are used to construct the 3D mesh quality prediction model, that is, BMQA-GSES. In the process of testing, the extracted features of the testing 3D meshes are inputted into the prediction model, which outputs the final quality score *Q*, expressed by

(19) $$Q=model(F_{S},F_{AM},F_{SI},F_{C},F_{DA},F_{A})$$

where *model* () is the quality prediction model trained by RF.

## 4. Experimental Results and Discussion

**Figure 11:**
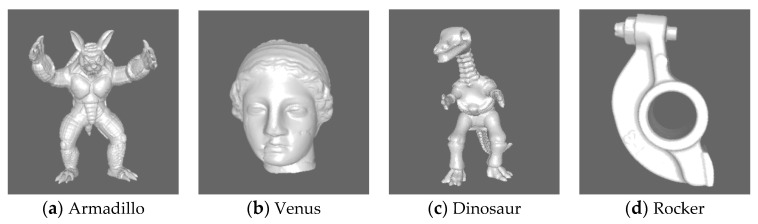
Reference 3D meshes of LIRIS/EPFL general-purpose database.

To verify the effectiveness of the proposed BMQA-GSES method, a series of experiments were performed in the public LIRIS/EPFL general-purpose database [11], which has been widely used for performance testing of 3D MQA methods. The LIRIS/EPFL general-purpose database consists of 88 3D meshes, including 4 reference 3D meshes and the corresponding 84 distorted 3D meshes. Every 3D mesh consists of 40,000 to 50,000 vertices and 80,000 to 90,000 triangular meshes. The reference 3D meshes are Armadillo, Venus, Dinosaur and Rocker, respectively, as shown in Figure 11. The distorted 3D meshes contain two types of distortion: random noise and smooth distortion. The subjective MOS of each distorted 3D mesh are available online, ranging from 0 (best quality) to 10 (worst quality). 

The proposed BMQA-GSES method has been compared with some influential and effective FR, RR and NR methods in our experiments. According to the standards recommended by Recommendation ITU-T P.1401 [32], the Pearson linear correlation coefficient (*r_p_*) and the Spearman rank order correlation coefficient (*r_s_*) are selected as the criteria to measure the performance of the proposed method (BMQA-GSES). Among them, *r_p_* can reflect the linear correlation between subjective and objective scores, while *r_s_* can reflect the consistency between subjective and objective scores. For an excellent objective MQA method, *r_p_* and *r_s_* should all be close to 1. In this paper, we randomly divide the database randomly into two 3D mesh sets, 80% of data is used for training and the other 20% is used for testing. To eliminate performance bias, random selection of the training and testing sets is repeated 1000 times, and the median performance indices for cases are adopted as the final results.

### 4.1. Graph Spectral and Spatial Features Analysis

In the proposed method, the graph spectral and spatial features are considered. The signal smoothness and the information entropy of amplitude features constitute the graph spectral feature, i.e., [*F_S_*, *F_AM_*], and shape index, curvedness, dihedral angle and the distribution triangular topological area constitute the spatial feature, i.e., [*F_SI_*, *F_C_*, *F_DA_*, *F_A_*]. Although the promising performance of the proposed quality evaluation model has been proved, the specific role of different feature is unkown. Therefore, it is necessary to analyze the performance of each feature separately in the paper. For this purpose, the performance results for every feature when used alone to learn the regression model are shown in Table 2.

From Table 2, the following observations can be derived. Firstly, graph spectral features include signal smoothness *F_S_* and the information entropy of amplitude *F_AM_*. Signal smoothness can reveal the intrinsic structure of graph signal to some extent. The 3D mesh with higher smoothness tends to be rougher, while the 3D mesh with lower smoothness tends to be smoother. In addition, the experimental results also show that the signal smoothness feature has good performance and overcomes the defect that the spatial signal cannot reflect the correlation and similarity between the vertices of the 3D mesh. Secondly, the Gaussian curvature signal is transformed by GFT in this paper. The Gaussian curvature in spatial domain can reflect the concave and convex information of the 3D mesh, but it cannot reveal the frequency information. Therefore, the information entropy of amplitudes under different frequency components are extracted in the graph spectral domain which have been proved that can represent the distortion of the 3D mesh, i.e., the low-frequency signal can represent the distortion information of the smoother region of the 3D mesh, and the high-frequency signal can represent the distortion information of the rough region of the 3D mesh. The experimental results show that the information entropy of amplitude features extracted in this paper can better reflect the surface distortion of the model. In addition, the performance of the method is improved to a certain extent when combining another graph spectral feature. Thirdly, in the case of considering the graph spectral features, some excellent spatial features are also combined in this paper. Shape index and curvedness can simulate human subjective visual perception information in spatial domain, i.e., concave and convex information of 3D mesh, while the dihedral angle of 3D mesh and the distribution of the triangular topology area can reflect the structure of 3D mesh, i.e., dihedral angle information and the distribution of the triangular topology area can reflect the change of the topology. Although any of the spatial features can play an important role alone, the ingenious combination of the four features can achieve better performance in the spatial domain. Finally, we observed that although the quality can be predicted well by using the features of the graph spectral domain or the spatial domain alone, the performance is further improved when all features are combined. This makes us believe that the graph spectral features and the spatial features are complementary to each other and should be simultaneously considered for objective quality assessment of 3D meshes.

### 4.2. Overall Performance Comparison

**Figure 12:**
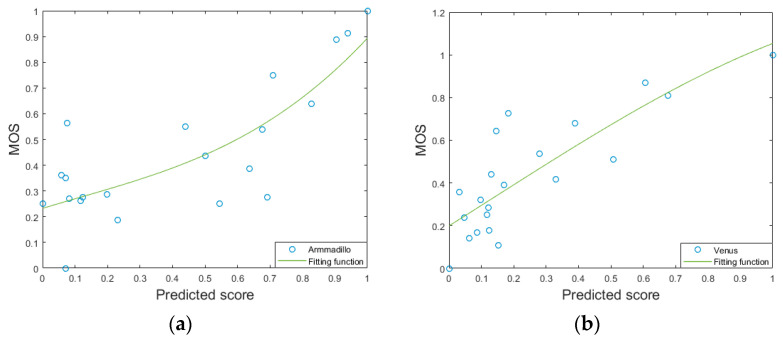

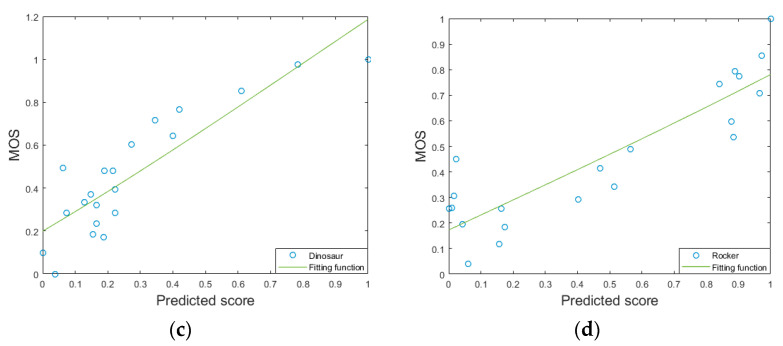
Scatter plots of each type the 3D mesh. (**a**) Armadillo, (**b**) Venus, (**c**) Dinosaur, (**d**) Rocker.

The proposed method is compared with some other state-of-the-art methods on the LIRIS/EPFL general-purpose database and the LIRIS masking database, as listed in Table 3. Table 3 describes the principles used by all compared methods. HD [6], GL2 [8], MSDM [11], MSDM2 [12] and DAME [13] are the FR MQA methods, 3DWPM1 [9], 3DWPM2 [10], FMPD [14], KLD Gamma [15] KLD Weibull [15] are the RR MQA methods, and NR-SVR [16] is the NR MQA method. The results of different methods are presented in Table 4. From Table 4, we make the following observations. Firstly, the classical methods such as HD, which are based on geometric distances, generally do not predict the visual quality of 3D mesh well, and they also ignore human visual perception characteristics. Secondly, the proposed method in this paper shows good performance for the most independent models and the whole database, especially for the whole database, the performance of the proposed method is obviously better than that of other methods. Figure 12 shows the scatter diagrams of the distorted 3D meshes corresponding to each geometry model that are used as testing 3D meshes. For example, in Figure 12a, the testing set is composed of all of the Armadillo’s distorted 3D meshes, while all the remaining distorted 3D meshes make up the training set. From the figures, it can be seen that the Prediction-MOS pairs are fitted well by the logistic function. However, most of the MQA methods in Table 4 are the FR MQA methods which require the original 3D mesh as the reference. By contrast, the proposed BMQA-GSES method is a blind one which potentially has wider application scopes. Thirdly, most existing 3D MQA methods only consider the characteristics of the spatial domain, while the proposed method, BMQA-GSES, takes into account the characteristics of other domains to describe the distortion information more comprehensively. The experimental results also show that the combined features of different transform domains achieve better performance. Fourthly, both proposed method and the method NR-SVR [16] perform better than the FR and RR MQA methods, although they are the blind methods. This phenomenon conveys an important message that it is possible to achieve better performance by blind MQA methods than those FR MQA ones. Finally, with respect to the blind MQA methods, the proposed BMQA-GSES method delivers higher *r_p_* and *r_s_* values than the method in NR-SVR [16].

The proposed method includes 13-dimensional graph spectral and spatial features, which are complementary with each other to better evaluate the visual quality of 3D mesh. Different from the most methods that use linear fitting pooling strategy, Random forest (RF) is adopted as the pooling strategy in our study. RF is a machine-learning algorithm with fast training speed and not easy to overfitting with a good prediction performance. However, the machine-learning pooling strategy will take a bit longer time than ordinary linear fitting pooling strategy. Although our method takes a little more time, the proposed method is highly desirable for the following reasons. Firstly, the performance of the proposed method is obviously better than other methods. Secondly, the proposed method in this paper is blind although most of the existing 3D MQA methods are mainly FR. It is very rare to be able to propose a blind 3D MQA method with such good prediction performance and the blind 3D MQA method is more widely used than the FR MQA method in practice. Thirdly, the new graph spectral features are extracts by GSP theory in the paper to describe the characteristics of the distortion 3D mesh, which can provide a new perspective for subsequent exploration of better 3D MQA methods.

## 5. Conclusions

The graph spectral features of three-dimensional (3D) mesh can reveal underlying shape information, while the spatial features of 3D mesh can simulate the external information of the 3D mesh that can be directly perceived by human eyes. Therefore, a new Blind Mesh Quality Assessment method based on Graph Spectral Entropy and Spatial features, called as BMQA-GSES, has been proposed based on the analysis of features in graph spectral domain and spatial domain. For graph spectral feature analysis, graph signal processing (GSP) is used to extract the signal smoothness and the information entropy of amplitudes under different frequency components to characterize the distortion of 3D mesh. For spatial feature analysis, the features of shape index and the curvedness are used to measure the concave and convex characteristic of the 3D mesh, the features of dihedral angles and the distribution of the triangular topology structure of 3D mesh are used to measure structural characteristic of 3D mesh. In addition, four spatial features are combined to characterize visual distortion of 3D mesh. All the extracted features are fused by the random forest regression to predict the objective quality score of the 3D mesh. The experimental results obtained on the public databases indicate the good performance of the method BMQA-GSES. However, there are some aspects of the proposed BMQA-GSES method requiring further study. For example, there is a lot of effective information that can be used in graph spectral domain. However, only the signal smoothness and the information entropy of amplitudes information are considered in the proposed method, thus, how to combine other useful information, such as phase, to describe the distorted of 3D mesh is an important problem that can be further studied in the future. In addition, GSP analysis tool Graph Fourier transform (GFT) is used to transform the signal from graph spectral domain to spatial domain in this paper. Therefore, whether there are other available GSP analysis tools (such as DCT) that can be used to describe the relevant information in other transform domains for evaluating the quality of 3D meshes is also an important issue that needs further study in the future.

## Figures and Tables

**Table 1:** Comparison of the mean shape index.

	Noise			Smooth		
**Level**	**Low**	**Medium**	**High**	**Low**	**Medium**	**High**
**Armadillo**	−0.2032	−0.1828	−0.1616	−0.2480	−0.2678	−0.2798
**Venus**	−0.1742	−0.1505	−0.1318	−0.2292	−0.2505	−0.2594
**Dinosaur**	−0.2655	−0.2456	−0.2233	−0.2813	−0.2815	−0.2836
**Rocker**	−0.0691	−0.0656	−0.0622	−0.0837	−0.0859	−0.0878

**Table 2:** Performance of different features on the LIRIS/EPFL general-purpose database (%).

Feature Types	Features	Armadillo		Venus		Dinosaur		Rocker		Whole Database	
		*r_p_*	*r_s_*	*r_p_*	*r_s_*	*r_p_*	*r_s_*	*r_p_*	*r_s_*	*r_p_*	*r_s_*
**Graph spectral domain**	***F_S_***	98.2	80.0	89.2	73.8	94.9	80.0	97.3	94.9	79.4	52.6
	***F_AM_***	97.0	80.0	90.8	80.0	71.9	40.0	98.3	80.0	94.8	62.4
**Spatial domain**	***F_SI_***	90.7	40.0	96.5	80.0	90.6	73.8	96.4	94.9	40.4	23.1
	***F_C_***	98.1	80.0	95.2	80.1	96.7	80.0	97.5	80.0	83.9	57.3
	***F_DA_***	98.6	80.0	98.5	80.0	98.1	75.5	93.5	94.8	84.9	87.0
	***F_A_***	96.5	79.9	94.5	79.9	98.2	80.0	96.9	80.1	82.2	86.6
**All**		**98.7**	**80.0**	**98.8**	**80.1**	**99.2**	**80.4**	**99.5**	**99.9**	**90.5**	**87.9**

**Table 3:** List of 3D MQA methods in the experiments.

Type	Method	Principle
**FR**	**HD** [6]	Hausdorff distance
	**GL2** [8]	vertex coordinate positions and the Geometrical Laplacian operator
	**MSDM** [11]	Local curvature, Contrast, and Structure
	**MSDM2** [12]	Multiscale mesh structural distortion
	**DAME** [13]	Dihedral angles
**RR**	**3DWPM1** [9]	Global roughness
	**3DWPM2** [10]	Global roughness
	**FMPD** [14]	Local roughness analysis and global roughness computation
	**KLD Gamma** [15]	Dihedral angles and statistical Gamma distributionmodel
	**KLD Weibull** [15]	Dihedral angles and statistical Weibull distribution model
**NR**	**NR-SVR** [16]	Dihedral angles, visual masking modulation and parameters estimation
	**The proposed**	Graph spectral features and spatial features

**Table 4:** Overall performance comparison on the LIRIS/EPFL general-purpose database (%).

Type	Method	Armadillo		Venus		Dinosaur		Rocker		Whole Database	
		*r_p_*	*r_s_*	*r_p_*	*r_s_*	*r_p_*	*r_s_*	*r_p_*	*r_s_*	*r_p_*	*r_s_*
**FR**	**HD** [6]	30.2	69.5	0.8	1.6	22.6	30.9	5.5	18.1	11.4	13.8
	**GL2** [8]	55.5	77.8	77.6	**91.0**	12.5	30.6	17.1	29.0	42.4	39.3
	**MSDM** [11]	70.0	**84.8**	72.3	87.6	56.8	73.0	75.0	89.8	75.0	73.9
	**MSDM2** [12]	85.3	81.6	87.5	89.3	85.7	85.9	87.2	89.6	81.4	80.4
	**DAME** [13]	76.3	60.3	83.9	**91.0**	88.9	**92.8**	80.1	85.0	75.2	76.6
**RR**	**3DWPM1** [9]	35.7	65.8	46.6	71.6	35.7	62.7	53.2	87.5	61.8	69.3
	**3DWPM2** [10]	43.1	74.1	16.4	34.8	19.9	52.4	29.9	37.8	49.6	49.0
	**FMPD** [14]	83.2	75.4	83.9	87.5	88.9	89.6	84.7	88.8	83.5	81.9
	**KLD Gamma** [15]	77.7	71.1	83.4	88.6	70.6	67.9	57.5	78.7	74.0	71.6
	**KLD Weibull** [15]	77.2	67.5	75.4	86.1	70.6	71.3	70.4	77.0	74.1	71.7
**NR**	**NR-SVR** [16]	91.5	76.8	88.6	85.7	84.1	78.6	86.6	86.2	87.8	81.5
	**The proposed**	**98.7**	80.0	**98.8**	80.1	**99.2**	80.4	**99.5**	**99.9**	**90.5**	**87.9**
